# CCL27: Novel Cytokine with Potential Role in Pathogenesis of Multiple Sclerosis

**DOI:** 10.1155/2015/189638

**Published:** 2015-07-29

**Authors:** Svetlana F. Khaiboullina, Aigul R. Gumerova, Irina F. Khafizova, Ekaterina V. Martynova, Vincent C. Lombardi, Saverio Bellusci, Albert A. Rizvanov

**Affiliations:** ^1^Institute of Fundamental Medicine and Biology, Kazan Federal University, Kazan, Tatarstan 420008, Russia; ^2^Department of Biochemistry and Molecular Biology, University of Nevada School of Medicine, Reno, NV 89557, USA; ^3^WP Institute, Reno, NV 89557, USA; ^4^Kazan State Medical University, 49 Butlerova Street, Kazan, Tatarstan 420012, Russia; ^5^Excellence Cluster Cardio-Pulmonary System, Justus Liebig University, Aulweg 130, 35392 Giessen, Germany

## Abstract

Multiple sclerosis (MS) is an autoimmune and neurodegenerative disease of unknown etiology. Leukocyte infiltration of brain tissue and the subsequent inflammation, demyelination, axonal damage, and formation of sclerotic plaques is a hallmark of MS. Upregulation of proinflammatory cytokines has been suggested to play an essential role in regulating lymphocyte migration in MS. Here we present data on serum cytokine expression in MS cases. Increased serum levels of IL-17 and IL-23 were observed, suggesting activation of the Th17 population of immune effector cells. Additionally, increased levels of IL-22 were observed in the serum of those with acute phase MS. Unexpectedly, we observed an upregulation of the serum chemokine CCL27 in newly diagnosed and acute MS cases. CCL27 is an inflammatory chemokine associated with homing of memory T cells to sites of inflammation. Therefore, its upregulation in association with MS suggests a potential role in disease pathogenesis. Our data supports previous reports showing IL-17 and -23 upregulation in association with MS and potentially identify a previously unknown involvement for CCL27.

## 1. Introduction

Multiple sclerosis (MS) is a chronic inflammatory disease of the central nervous system (CNS) with undefined etiology. The disease has a polysymptomatic onset and is usually first diagnosed between the ages of 20–40 years [1]. Although there are no clinical findings unique to MS, some symptoms are characteristic of the disease, such as sensory abnormalities and visual and motor impairment [2]. In 80–85% of cases, initial progression of MS is characterized by episodes of neurological disability and recovery. This clinical presentation is classified as remitting-relapsing MS (RRMS) [3, 4]. As the disease progresses, 60–70% of RRMS cases will gradually worsen with a steady progression of symptoms [5]. This pattern of disease is referred to as secondary RRMS. A small group of cases (approximately 10%) develop MS characterized with a steady progression of neurological symptoms without periods of recovery. This is classified as primary-progressive MS (PPMS) [6, 7].

Formation of multiple brain lesions is a typical characteristic of MS. Sclerotic plaques form at the sites of inflammation, demyelination, and axonal damage. It is believed that autoreactive T lymphocytes play a major role in initiating the immune assault against axonal myelin sheets in the CNS, which leads to demyelination and subsequent neuronal death [5, 8]. Increased permeability of the blood brain barrier (BBB), as documented in MS cases [9–11], is essential for leukocyte migration into brain tissue. Accordingly, previous studies have shown substantial remodeling of the BBB in MS cases. For example, decreased expression of tight junction molecules in BBB endothelial cells has been observed in MS [11, 12]. Disruption of BBB integrity is further confirmed by the observation of elevated matrix metalloprotease (MMP) 2 and 9 expression in MS brain lesions [13–15]. Similarly, upregulation of MMP2 and 9 has been shown in astrocytes and neuroglia, implicating these cells in the pathogenesis of MS [13, 14].

Leukocyte infiltration is a common finding at the sites of MS brain lesions. Previous studies have reported high IFN-*γ* secretion by autoreactive T lymphocytes, suggesting a Th1 phenotype of myelin-specific T cells [16–18]. Recently, a Th17 lymphocyte subset was also shown to play a role in MS pathogenesis. In this study, Brucklacher-Waldert et al. reported an increase in the numbers of Th1 and Th17 cells in the blood and cerebrospinal fluid (CSF) of MS cases [19]. They also reported a significant increase in the number of Th17 lymphocytes during the relapse stage; however, Th1 counts remained unchanged [19]. Furthermore, they determined that Th17 cells from MS cases had a higher proliferative capacity and were less susceptible to suppression, as compared to Th1 cells. In another study, Kebir et al. demonstrated that, in MS, Th17 lymphocytes more readily cross the BBB as compared to Th1 cells [20]. Therefore, they suggested that Th17 lymphocytes might be more encephalitogenic than Th1 cells.

A compromised BBB and facilitated migration of autoreactive immune effector cells are essential for development of MS. Both BBB integrity and leukocyte trafficking are regulated by cytokines. Previous studies have shown upregulation of Th1 type cytokines, such as IL-2, IFN*γ*, and IL-12, in subjects with MS, while the Th2 cytokines, IL-4 and IL-10, were downregulated [21, 22]. Furthermore, administration of IFN*γ* exacerbated clinical and hematological symptoms of MS [23, 24], and higher levels of IL-2 and lower levels of IL-10 have been detected in relapsed MS cases [21]. Elevated levels of IL-17 have been observed in the CSF and serum of MS cases [25], and during clinical exacerbations, higher levels of IL-17 mRNA were also detected in the CSF relative to that in the blood [26]. Subsequently, Th17 transcripts were detected in MS lesions [25]. IL-17-producing leukocytes have been suggested to belong to a new subset of Th17 lymphocytes that is maintained and driven by IL-23 [27, 28]. Therefore, the current paradigm of MS pathogenesis indicates that Th17 lymphocytes together with Th1 cells are central to development of neuroinflammation, demyelination, and neural death [29, 30].

Although it is well established that cytokines play a central role in lymphocytes breaching the BBB, as well as their subsequent migration into neuronal tissue, our knowledge regarding cytokine activation and their involvement in MS is limited. Here we report data on serum levels of 57 cytokines in MS cases with different clinical presentations. Overall, our data support a role for mononuclear leukocytes in the pathogenesis of MS. Furthermore, our data support the previous observations of others regarding the upregulation of IL-17 group cytokines in MS, thus providing conformational evidence of their involvement in MS pathogenesis. We observed upregulated levels of serum IL-22 in acute stage RRMS, suggesting that Th22 lymphocytes may play a role during MS exacerbations. We also observed that serum CCL27 was upregulated in MS cases. To the best of our knowledge, this report is the first to describe a CCL27 involvement in association with MS.

## 2. Materials and Methods

### 2.1. Study Subjects and Samples

A total of 42 cases were admitted to the Department of Neurology, Neurosurgery and Medical Genetics of Kazan State Medical University, Russian Federation. A diagnosis of MS was established based upon clinical presentation and brain MRI scans. Serum from 20 healthy individuals was collected to serve as controls. Informed consent was obtained from each subject according to the clinical and experimental research protocol, approved by the Local Ethic Expert Committee of the Kazan State Medical University (number 196, 10 May 2010).

### 2.2. Cytokine Analysis

Serum cytokine levels were analyzed using Bio-Plex (Bio-Rad, Hercules, CA, USA) multiplex magnetic bead-based antibody detection kits following the manufacturer's instructions. Bio-Plex Pro Human Th17 Cytokine Panel, Bio-Plex Pro Human Cytokine 27-plex Panel, and Bio-Plex Human Cytokine 21-plex Panel were used for detection of a total of 57 analytes. Serum aliquots (50 *μ*L) were collected from healthy donors and 42 MS cases. A minimum of 50 beads per analyte was acquired. Median fluorescence intensities were measured using a Luminex 200 analyzer. Data collected was analyzed with MasterPlex CT control software and MasterPlex QT analysis software (Hitachi Software San Bruno, CA, USA). Standard curves for each analyte were generated using standards provided by manufacturer.

### 2.3. Statistical Analysis

Statistical analysis was conducted using Statistica and XLSTAT software (StatsSoft, Tulsa, OK and Addinsoft, New York, NY, resp.). Differences between the means of compared groups were analyzed using the Mann-Whitney test for nonparametric data with significance at *P* ≤ 0.05.

## 3. Results

### 3.1. Patients

Serum samples from 42 MS cases (40 female and 2 male) were analyzed. MS diagnosis was established according to the 2010 Revised Diagnostic Criteria for MS [31]. Thirty-two (76.2%) cases were diagnosed with RRMS, 7 (16.7%) were diagnosed with secondary RRMS, and 3 (7.1%) were newly diagnosed. The mean age for MS cases was 41.6 years (24–58 years) and mean duration of the disease was 12.4 years (1–32 years). Expanded Disability Status Scale (EDSS) score was 3.5 (0–10). MRI revealed multiple lesions in the subcortical region, corpus callosum, and pons. In some subjects, demyelination foci were detected in cervical and thoracic regions of the spinal cord. Five subjects received glatiramer, while the remaining 38 receive no treatment.

### 3.2. Cytokine Profile

A total of 57 cytokines were analyzed. Serum levels of 9 cytokines, IL12-p40, CCL27, M-CSF, MIF, IL-17A, IL-23, CCL2, CCL3, and IL-2Ra, differed significantly between the serum of all MS cases and the controls (Table 1). On average, serum levels of HGF, TRAIL, CXCL10, and CCL5 were elevated in MS cases; however, they did not reach statistical significance when compared to control sera. Serum levels of the remaining 42 cytokines were generally similar between cases and controls (Table 1).

**Figure 1:**
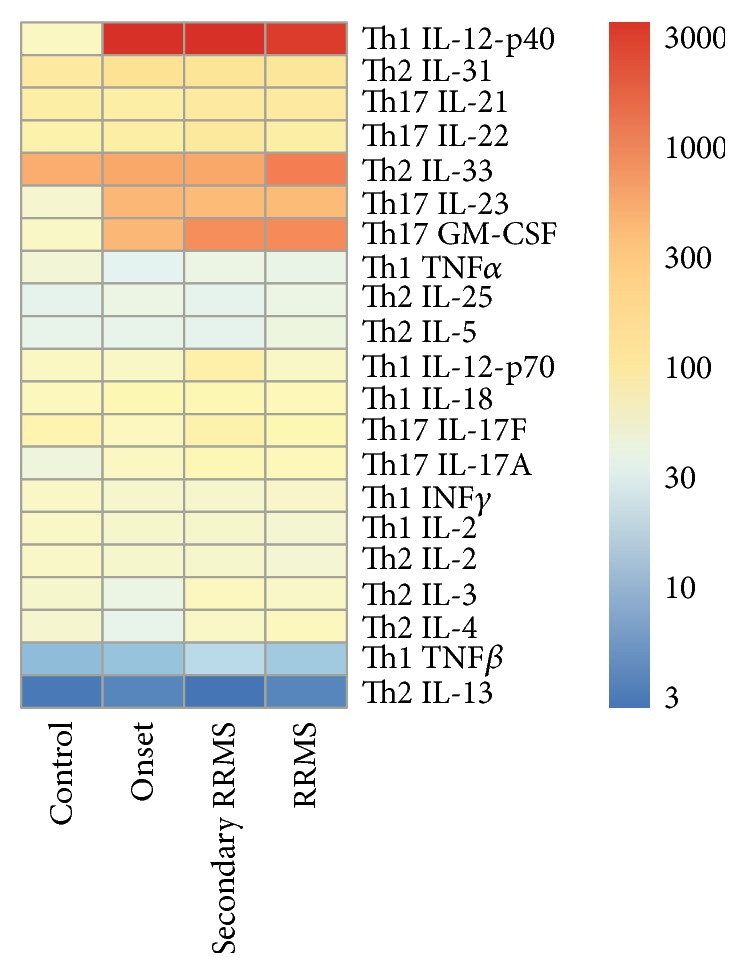
Heat map analysis of serum cytokine profile in MS cases. All MS cases were grouped based on presentation/stage of the disease. Serum cytokine profile in newly diagnosed cases (onset), secondary RRMS, and RRMS forms of MS were analyzed.

To further elucidate the role of cytokines in MS, we organized the respective cytokines by functionality. Three categories of cytokines were investigated based on their putative role in immune activation. Cytokines that activate Th1 immune effector cells made of the first group and included IL-2, IL-12, and IFN*γ*, while Th2 cytokines, which included IL-4, IL-5, IL-9, IL-10, and IL-13, made up the second group. The final group of cytokines included those that activate Th17 lymphocytes and were IL-6, IL-23, IL-1*β*, IL-17a, IL-21, and IL-22. Among the Th1 cytokines, only serum IL-12p40 was significantly upregulated in MS cases when compared to controls (Table 1). Similarly, heat map analysis revealed upregulation of IL-12p40 in all MS cases as compared to healthy controls (Figure 1). Serum levels of IL-2 and IFN*γ* remained unchanged in MS cases and no differences were observed in the levels of Th2 cytokines between MS cases and controls (Table 1, Figure 1). However, serum levels of the Th17 cytokines IL-17a and IL-23 were significantly higher in MS cases as compared to controls (Table 1). Likewise, heat map analysis has shown increased serum concentration of IL-23 and IL-17a (Figure 1).

Increased IL-12p40, CCL2, and M-CSF in the serum of MS cases suggest activation of mononuclear immune effector cells. Additionally, serum levels of CCL27 were significantly increased in MS cases when compared to healthy controls. CCL27 is a well-known chemoattractant for mononuclear leukocytes and has been shown to attract memory T cells to the site of cutaneous lesions [32]. Therefore, when considered together, our data suggests that the serum cytokine profile of subjects with MS is characteristic of a classic Th17/Th1 shift which promotes mononuclear leukocytes infiltration of inflamed tissue.

**Figure 2:**
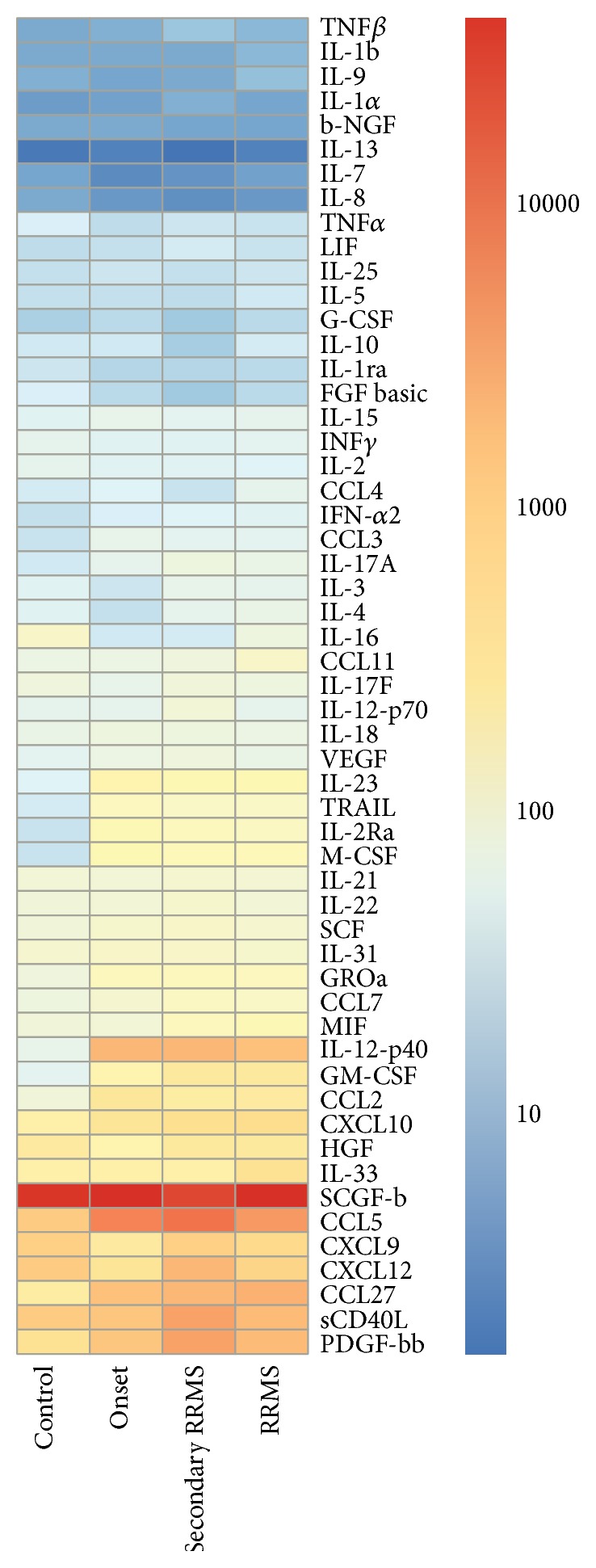
Heat map analysis of serum cytokines in MS cases with different presentation/stage of the disease. All MS cases were grouped based on presentation/stage of the disease. Serum cytokine profile in newly diagnosed cases (onset), secondary RRMS, and RRMS forms of MS were analyzed.

Finally, we analyzed the serum cytokine profile of MS cases with respect to the different clinical presentations. Cases were organized into 3 groups: those with secondary RRMS acute phase, those with RRMS in remission, and those who were newly diagnosed with MS. Acute phase secondary RRMS was characterized by significant changes in the serum levels of 13 cytokines, while changes in smaller number of cytokines were detected in the serum of RRMS cases in remission and newly diagnosed MS (Table 2, Figure 2). Further analysis revealed that serum IL-12(p40), M-CSF, CCL2, and IL-23 were significantly upregulated in all MS cases, regardless of the stage or phase of the disease (Table 3, Figure 2). All MS cases were characterized by an increase in serum levels of GROa (CXCL1), CCL7, and IL-22; however, these changes were only significant for acute phase secondary RRMS cases. Interestingly, subjects with acute phase secondary RRMS had significantly higher level of serum CCL27 and TRAIL, similar to that observed in newly diagnosed MS cases. However, newly diagnosed MS cases had significantly lower levels of serum XCL9, CXCL12, and CCL3, while the levels of these cytokines in all RRMS cases did not differ from controls. Upregulation of IL-2Ra, MIF, and IL-17A was observed for all RRMS cases; however, these cytokines did not significantly differ between newly diagnosed MS cases and controls (Table 2, Figure 2).

## 4. Discussion

Lymphocyte infiltration of brain tissue, demyelination, and gliosis are hallmarks of MS. It is believed that brain infiltrating autoreactive lymphocytes promote axonal demyelination leading to neuronal death and sclerotic plaque formation. A subset of Th17 lymphocytes has been implicated in the pathogenesis of MS. For example, high levels of IL-17 cytokine have been observed in serum and CSF of MS cases [25]. Also, adoptive transfer of IL-17-producing T lymphocytes induced experimental autoimmune encephalitis (EAE), an experimental model of MS [28], and administration of anti-IL17 antibodies prevented development of EAE and delayed the onset of symptoms [28, 33]. Clinical studies have reported the presence of IL-17 and IFN-*γ* expressing T lymphocytes in the brain tissue of RRMS cases [20]. Additionally, high levels of IL-17 mRNA have been detected in blood and CSF of MS cases, with the highest levels observed during exacerbations [26]. Our data supports a role for IL-17 in the pathogenesis of MS. We have observed significantly increased levels of IL-17 in serum of RRMS cases. Interestingly, levels of IL-17 in serum of newly diagnosed MS were also elevated, although differences were not significant compared to controls. These data suggest that Th17 activation occurs early during MS and increases with progression of the disease.

IL-23 has been shown to play role in the activation of Th17 type immunity. Current evidence suggests that IL-23 has an essential function in the differentiation and expansion of Th17 T lymphocytes from naïve CD4+ T cells [27, 33, 34]. Therefore it is believed that IL23-IL17 axis plays an important role in developing autoimmunity [35, 36]. IL-23 shares the p40 subunit with IL-12, another cytokine implicated in MS pathogenesis [37, 38]. Furthermore, it has been shown that IL-23, rather than IL-12, is required for the development of the EAE [39–41]. Another important function of IL-23, as demonstrated by Langrish et al., is to promote the expansion of encephalitogenic T cells that drive the production of IL-17A and IL-17F [28]. Our data suggests that IL-23 is upregulated in the serum of all MS cases, regardless of the stage of the disease. The fact that increased serum IL-23 levels did not depend of the progression of the disease suggests an essential role of IL-23 in establishing and maintaining autoimmunity.

We observed increased levels of serum CCL27 for all MS cases; however, the differences reached statistical significant only for the RRMS cases. CCL27 upregulation is primarily associated with the pathogenesis of atopic dermatitis [42]; however, it is likely that its role is not exclusively restricted to skin inflammation. For instance, the enhancement of mucosal immunity has been demonstrated in animals immunized with plasmids containing HIV gag and CCL27 [43]. Furthermore, expression of CCL27 mRNA has been detected in brain tissue, particularly the cerebral cortex and limbic structures [44]. Expression of CCR10, the receptor for CCL27, has been confirmed in astrocytes and neurons of the hippocampus [44–46]. Additionally, CCL27 acts as chemoattractant for antigen-specific T lymphocytes [47]. This suggests that CCL27 may facilitate autoreactive T lymphocyte migration into brain tissue of MS cases, thus promoting brain inflammation.

Our data suggest that IL-22 is upregulated in the serum of acute secondary RRMS. Current knowledge regarding IL-22 expression in association with MS is limited as only a small number of reports address its role in the pathogenesis of autoimmune demyelinating diseases. Almolda and coworkers reported that changes in serum IL-22 levels correlate with the development of EAE [48]. They further reported that serum IL-22 levels increase during the acute phase, peak at the height of clinical presentation, and decrease during recovery. Recently, increased plasma and CSF levels of IL-22 have been reported for subjects diagnosed with Guillain-Barré syndrome, another acute autoimmune-mediated inflammatory demyelinating disease [49]. Li et al. reported that IL-17 and IL-22 levels in CSF correlate with disease disability [49]. Our data suggest that serum levels of IL-22 are significantly elevated during the acute phase of secondary RRMS when compared to healthy controls. Although it is slightly elevated during the remission phase of RRMS, serum IL-22 did not significantly differ from controls. These data are consistent with the observation of Almolda et al. whereby changes in serum IL-22 reflect the stage of the disease [48]. IL-22 is produced by variety of leukocytes including Th17 cells [50]. Although IL-22 and IL-17 are often simultaneously present at high levels in inflamed tissues, their biological effects differ. For example, IL-22 primarily activates an innate immune response, while IL-17 typically acts as a proinflammatory mediator [51–54]. This suggests that the MS cytokine milieu promotes chronic inflammation and activation of immune effector cells.

In summary, our data provide evidence that mononuclear leukocytes play a role in the pathogenesis of MS. The serum cytokine profiles observed in our MS study subjects suggest Th17 activation, consistent with the previous reports of others [25, 28, 33]. Furthermore and adding to the present body of knowledge, we observed an increase in serum IL-22 during the acute phase of MS. We also observed a general upregulation of CCL27 in association with all presentations of MS. Previous studies regarding the role of CCL27 in human pathology are primarily limited to atopic dermatitis [55]; however, our data support a broader function involving MS inflammation and lymphocyte activation. Future studies will be required to delineate the role of CCL27 in MS.

## Figures and Tables

**Table 1:** Serum cytokine levels in MS cases.

Analyte	MS (ng/mL)	Control (ng/mL)
IL-1*α*	6.6 ± 0.8	5.3 ± 2.2
IL1b	8.8 ± 0.9	6.6 ± 0.4
IL-1ra	23 ± 7.3	38 ± 17.2
IL-2Ra	320.3 ± 51.9	36.3 ± 4.1
	*P* < **0.04**	
IL-2	56.23 ± 32	78 ± 27
IL-3	82.0 ± 12.9	67.2 ± 13.4
IL4	87.8 ± 15.4	63.0 ± 18.6
IL-5	24.12 ± 17	33 ± 23
IL-7	5.2 ± 0.3	6.2 ± 0.8
IL-8	4.7 ± 0.5	6.6 ± 1.6
IL-9	9.7 ± 1.7	7.3 ± 1.4
IL10	73.6 ± 6.2	68.5 ± 10.2
IL-12p40	4005.7 ± 327.1	86.0 ± 6.1
	*P* < **0.00015**	
IL-12(p70)	90.2 ± 17.9	84.1 ± 8.6
IL-13	2.7 ± 0.3	2.1 ± 0.2
IL-15	85.0 ± 3.4	68.6 ± 4.6
IL-16	119.7 ± 33.9	241.1 ± 67.9
IL17A	103.79 ± 6.5	44.74 ± 6.3
	*P* = **0.001**	
IL17F	115.2 ± 8.3	44.7 ± 6.3
IL-18	118.3 ± 12.0	101.9 ± 24.3
IL21	184.3 ± 6.5	162.005 ± 16.1
IL22	163.5 ± 7.7	149.358 ± 18.7
IL23	481.4 ± 29.8	62.1517 ± 8.9
	*P* < **0.000003**	
IL25	36.8 ± 1.9	32.4 ± 1.7
IL31	230.5 ± 14.2	195.055 ± 29.8
IL33	1050.4 ± 291.0	683.6 ± 158.2
CCL2	915.9 ± 46.9	145.6 ± 23.0
	*P* = **0.0000001**	
CCL3	73.8 ± 7.1	35.7 ± 4.1
	*P* < **0.05**	
CCL4	64.2 ± 8.9	47.5 ± 13.2
CCL5	8743.6 ± 2219.2	2589.5 ± 248.3
CCL7	337.4 ± 55.2	119.8 ± 6.3
CCL11	220.3 ± 25.0	113.5 ± 28.8
CCL27	4674.9 ± 643.3	854.6 ± 51.0
	*P* < **0.05**	
CXCL1	342.9 ± 46.2	135.6 ± 40.6
CXCL9	2307.1 ± 338.7	2461.8 ± 301.6
CXCL10	1804.3 ± 264.6	732.2 ± 111.5
CXCL12	2575.6 ± 355.1	2582.4 ± 166.5
IFN-*α*2	59.8 ± 6.7	33.5 ± 8.0
INF*γ*	74.1 ± 4.1	77.3 ± 8.9
FGF basic	34 ± 24	56 ± 26
G-CSF	26.3 ± 2.9	19.7 ± 4.3
GM-CSF	45 ± 21	76 ± 34
HGF	1206.7 ± 164.1	931.7 ± 287.7
LIF	44.0 ± 7.9	29.2 ± 14.5
M-CSF	430.2 ± 41.7	37.3 ± 7.0
	*P* = **0.002**	
MIF	401.1 ± 32.9	148.6 ± 38.9
	*P* = **0.01**	
b-NGF	6.6 ± 0.4	6.7 ± 0.8
PDGF-bb	1256 ± 235	1462 ± 562
sCD40L	3592.9 ± 620.7	2756.0 ± 1007.3
SCF	230.5 ± 25.8	142.7 ± 18.9
SCGF-b	51782.6 ± 8900.2	46927.3 ± 7531.6
TNF*α*	39.8 ± 3.5	55.0 ± 5.3
TNF-*β*	10.4 ± 1.4	7.0 ± 1.5
TRAIL	252.3 ± 41.7	46.2 ± 10.0
VEGF	114.6 ± 12	74.4 ± 10.4

**Table 2:** Cytokines and disease progression/stage.

Analyte	Secondary RRMS acute (ng/mL)	RRMS remission (ng/mL)	MS onset (ng/mL)	Control (ng/mL)
IL-1*α*	8.0 ± 1.8	6.09 ± 1.0	5.9 ± 1.0	5.3 ± 1.1
IL1*β*	6.8 ± 1.4	9.2 ± 1.2	7.1 ± 0.3	6.6 ± 0.4
IL-1ra	24.6 ± 12.5	27.3 ± 9.4	24.5 ± 12	38 ± 17.2
IL-2Ra	376.1 ± 71.7	301.5 ± 61.2	481.0 ± 303.5	36.3 ± 4.1
	P = **0.001**	P < **0.04**		
IL-2	67.3 ± 12.4	59.3 ± 23	67.3 ± 12	78 ± 27
IL-3	93.8 ± 15.8	78.7 ± 15.0	39.6 ± 9.6	67.2 ± 13.5
IL4	80.6 ± 25.9	95.9 ± 23.5	33.2 ± 5.0	63.0 ± 18.6
IL-5	30.5 ± 12.5	42.4 ± 23	33.2 ± 16.2	33 ± 23
IL-7	4.2 ± 0.6	5.5 ± 0.4	3.5 ± 0.03	6.2 ± 0.8
IL-8	3.6 ± 0.6	0.4 ± 0.7	4.7 ± 0.3	6.6 ± 1.6
IL-9	6.9 ± 1.4	11.0 ± 2.9	6.2 ± 0.6	7.3 ± 1.4
IL10	18.1 ± 4.2	49.8 ± 9.5	42.3 ± 16.6	45.4 ± 10.2
IL-12p40	3892.5 ± 821.9	3248.8 ± 327.7	4016.3 ± 571.7	86.0 ± 6.1
	P = **0.003**	P = **0.00014**	P = **0.00008**	
IL-12 (p70)	159.7 ± 103.2	78.0 ± 9.4	77.1 ± 0.7	84.1 ± 8.6
IL-13	1.9 ± 0.3	2.6 ± 0.3	2.7 ± 0.8	2.1 ± 0.2
IL-15	75.0 ± 6.5	80.4 ± 2.7	86.9 ± 11.2	68.6 ± 4.6
IL-16	51.0 ± 10.8	123.6 ± 51.7	42.0 ± 13.1	241.1 ± 67.9
	P = **0.01**			
IL17A	119.0 ± 12.0	104.3 ± 8.9	82.7 ± 28.0	44.7 ± 6.3
	P < **0.0003**	P = **0.0023**		
IL17F	145.7 ± 26.7	119.8 ± 10.7	88.5 ± 9.9	137.9 ± 30.4
IL-18	124.4 ± 21.5	104.9 ± 8.5	126.4 ± 38.0	101.9 ± 24.3
IL21	194.5 ± 17.0	191.1 ± 8.9	169.5 ± 24.6	162.0 ± 16.1
IL22	216.9 ± 15.0	162.6 ± 9.4	173.1 ± 28.8	149.4 ± 18.7
	P < **0.02**			
	^*^ *P* = **0.007**			
IL23	530.2 ± 84.5	527.3 ± 27.2	585.3 ± 180.9	62.2 ± 8.9
	P <**0.0004**	P = **0.000000002**	P = **0.003**	
IL25	31.9 ± 4.9	39.1 ± 2.8	40.7 ± 7.0	32.4 ± 1.7
IL31	253.8 ± 49.9	234.9 ± 19.5	262.5 ± 14.2	195.1 ± 29.8
IL33	717.2 ± 192.9	1370.7 ± 501.6	720.0 ± 221.0	683.6 ± 158.1
CCL2	821.1 ± 80.5	918.3 ± 54.8	1152.5 ± 192.5	145.6 ± 23.0
	P < **0.00002**	P = **0.00000001**	P < **0.005**	
CCL3	72.3 ± 18.2	73.1 ± 10.7	89.4 ± 10.8	35.7 ± 4.1
			P = **0.0002**	
CCL4	37.5 ± 10.7	82.7 ± 13.9	57.7 ± 25.9	47.5 ± 13.2
CCL5	15926.6 ± 11653.2	7726.7 ± 1699.9	11287.8 ± 4843.4	2589.5 ± 248.3
CCL7	317.0 ± 65.4	286.7 ± 63.6	211.2 ± 93.5	119.8 ± 6.3
	P = **0.03**			
CCL11	128.6 ± 31.3	246.7 ± 29.7	107.3 ± 14.6	113.5 ± 28.8
CCL27	4192.9 ± 773.6	4282.5 ± 912.8	3152.7 ± 582.9	854.6 ± 51.0
	P < **0.005**		P < **0.002**	
CXCL1	362.7 ± 64.1	332.0 ± 48.7	379.9 ± 194.6	135.6 ± 40.6
	P = **0.02**			
CXCL9	2319.4 ± 687.2	1878.1 ± 314.4	967.2 ± 536.4	2461.8 ± 301.6
			P = **0.04**	
CXCL10	1655.1 ± 80.5	1754.4 ± 384.4	1308.3 ± 500.6	732.2 ± 111.5
CXCL12	59.3 ± 13.3	65.4 ± 9.0	51.3 ± 15.9	33.5 ± 8.0
IFN-*α*2	68.8 ± 23.3	46.3 ± 12	79 ± 12	40.9 ± 16.4
INF*γ*	48.3 ± 12.3	67.3 ± 23	67.2 ± 12	56 ± 26
FGF basic	16.5 ± 4.0	27.5 ± 3.8	26.5 ± 6.9	19.7 ± 4.3
G-CSF	56.3 ± 12.3	66.4 ± 24	78.3 ± 6	76 ± 34
GM-CSF	1085.5 ± 299.7	1101.2 ± 149.4	570.5 ± 117.4	931.7 ± 287.7
HGF	48.3 ± 21.7	37.3 ± 5.0	32.0 ± 10.0	29.2 ± 14.5
LIF	438.7 ± 75.4	401.1 ± 43.3	495.6 ± 142.0	37.3 ± 7.0
	P = **0.001**	P = **0.00065**	P < **0.005**	
M-CSF	368.3 ± 53.2	462.2 ± 43.2	173.5 ± 15.8	148.6 ± 38.9
	P = **0.01**	P < **0.003**		
MIF	6.3 ± 0.7	6.3 ± 0.4	7.1 ± 1.6	6.7 ± 0.8
b-NGF	1324.5 ± 256.3	1526 ± 329.3	1134 ± 213	1462 ± 562
PDGF-bb	6012.3 ± 2966.4	3606.5 ± 592.3	2962.4 ± 906.1	2756.0 ± 1007.3
sCD40L	251.2 ± 51.1	206.9 ± 16.3	222.4 ± 65.9	142.7 ± 18.9
SCF	33385.6 ± 4425.1	54557.7 ± 13801.1	49672.2 ± 13153.3	46927.3 ± 7531.6
SCGF-b	4089.7 ± 1952.1	2174.5 ± 188.5	1254.8 ± 22.6	2582.4 ± 166.5
			P < **0.001**	
TNF*α*	39.4 ± 8.9	36.0 ± 4.6	28.6 ± 14.1	55.0 ± 5.3
TNF-*β*	13.8 ± 3.2	8.9 ± 1.5	7.9 ± 1.4	7.0 ± 1.5
TRAIL	283.1 ± 22.7	271.1 ± 72.0	332.5 ± 29.2	46.2 ± 1.5
	P = **0.000002**		P = **0.000006**	
VEGF	135.9 ± 43.4	97.4 ± 13.4	111.5 ± 39.1	74.4 ± 10.5

P: to control.

^*^P: to RRMS remission.

**Table 3:** MS serum cytokines differ from healthy controls.

Analyte	Secondary RRMS acute (ng/mL)	RRMS remission (ng/mL)	MS onset (ng/mL)	Control (ng/mL)
IL-2Ra	376.1 ± 71.7	301.5 ± 61.2	481.0 ± 303.5	36.3 ± 4.1
	*P* = **0.001**	*P* < **0.04**		
IL-12p40	3892.5 ± 821.9	3248.8 ± 327.7	4016.3 ± 571.7	86.0 ± 6.1
	*P* = **0.003**	*P* = **0.00014**	*P* = **0.00008**	
IL-16	51.0 ± 10.8	123.6 ± 51.7	42.0 ± 13.1	241.1 ± 67.9
	*P* = **0.01**			
IL17A	119.0 ± 12.0	104.3 ± 8.9	82.7 ± 28.0	44.7 ± 6.3
	*P* < **0.0003**	*P* = **0.0023**		
IL22	216.9 ± 15.0	162.6 ± 9.4	173.1 ± 28.8	149.4 ± 18.7
	*P* < **0.02**			
	^*^ *P* = **0.007**			
IL23	530.2 ± 84.5	527.3 ± 27.2	585.3 ± 180.9	62.2 ± 8.9
	*P* < **0.0004**	*P* = **0.000000002**	*P* = **0.003**	
CCL2	821.1 ± 80.5	918.3 ± 54.8	1152.5 ± 192.5	145.6 ± 23.0
	*P* < **0.00002**	*P* = **0.00000001**	*P* < **0.005**	
CCL3	72.3 ± 18.2	73.1 ± 10.7	89.4 ± 10.8	35.7 ± 4.1
			*P* = **0.0002**	
CCL7	317.0 ± 65.4	286.7 ± 63.6	211.2 ± 93.5	119.8 ± 6.3
	*P* = **0.03**			
CCL27	4192.9 ± 773.6	4282.5 ± 912.8	3152.7 ± 582.9	854.6 ± 51.0
	*P* < **0.005**		*P* < **0.002**	
CXCL1	362.7 ± 64.1	332.0 ± 48.7	379.9 ± 194.6	135.6 ± 40.6
	*P* = **0.02**			
CXCL9	2319.4 ± 687.2	1878.1 ± 314.4	967.2 ± 536.4	2461.8 ± 301.6
			*P* = **0.04**	
M-CSF	438.7 ± 75.4	401.1 ± 43.3	495.6 ± 142.0	37.3 ± 7.0
	*P* = **0.001**	*P* = **0.00065**	*P* < **0.005**	
MIF	368.3 ± 53.2	462.2 ± 43.2	173.5 ± 15.8	148.6 ± 38.9
	*P* = **0.01**	*P* < **0.003**		
CXCL12	4089.7 ± 1952.1	2174.5 ± 188.5	1254.8 ± 22.6	2582.4 ± 166.5
			*P* < **0.001**	
TRAIL	283.1 ± 22.7	271.1 ± 72.0	332.5 ± 29.2	46.2 ± 1.5
	*P* = **0.000002**		*P* = **0.000006**	
